# Deconvolution analysis identified altered hepatic cell landscape in primary sclerosing cholangitis and primary biliary cholangitis

**DOI:** 10.3389/fmed.2024.1327973

**Published:** 2024-05-15

**Authors:** Hoang Nam Pham, Linh Pham, Keisaku Sato

**Affiliations:** ^1^Department of Life Sciences, University of Science and Technology of Hanoi, Vietnam Academy of Science and Technology, Hanoi, Vietnam; ^2^Department of Science and Mathematics, Texas A&M University—Central Texas, Killeen, TX, United States; ^3^Division of Gastroenterology and Hepatology, Department of Medicine, Indiana University School of Medicine, Indianapolis, IN, United States

**Keywords:** deconvolution analysis, CIBERSORTx, primary sclerosing cholangitis, primary biliary cholangitis, hepatic cell landscape

## Abstract

**Introduction:**

Primary sclerosing cholangitis (PSC) and primary biliary cholangitis (PBC) are characterized by ductular reaction, hepatic inflammation, and liver fibrosis. Hepatic cells are heterogeneous, and functional roles of different hepatic cell phenotypes are still not defined in the pathophysiology of cholangiopathies. Cell deconvolution analysis estimates cell fractions of different cell phenotypes in bulk transcriptome data, and CIBERSORTx is a powerful deconvolution method to estimate cell composition in microarray data. CIBERSORTx performs estimation based on the reference file, which is referred to as signature matrix, and allows users to create custom signature matrix to identify specific phenotypes. In the current study, we created two custom signature matrices using two single cell RNA sequencing data of hepatic cells and performed deconvolution for bulk microarray data of liver tissues including PSC and PBC patients.

**Methods:**

Custom signature matrix files were created using single-cell RNA sequencing data downloaded from GSE185477 and GSE115469. Custom signature matrices were validated for their deconvolution performance using validation data sets. Cell composition of each hepatic cell phenotype in the liver, which was identified in custom signature matrices, was calculated by CIBERSORTx and bulk RNA sequencing data of GSE159676. Deconvolution results were validated by analyzing marker expression for the cell phenotype in GSE159676 data.

**Results:**

CIBERSORTx and custom signature matrices showed comprehensive performance in estimation of population of various hepatic cell phenotypes. We identified increased population of large cholangiocytes in PSC and PBC livers, which is in agreement with previous studies referred to as ductular reaction, supporting the effectiveness and reliability of deconvolution analysis in this study. Interestingly, we identified decreased population of small cholangiocytes, periportal hepatocytes, and interzonal hepatocytes in PSC and PBC liver tissues compared to healthy livers.

**Discussion:**

Although further studies are required to elucidate the roles of these hepatic cell phenotypes in cholestatic liver injury, our approach provides important implications that cell functions may differ depending on phenotypes, even in the same cell type during liver injury. Deconvolution analysis using CIBERSORTx could provide a novel approach for studies of specific hepatic cell phenotypes in liver diseases.

## 1 Introduction

Primary sclerosing cholangitis (PSC) and primary biliary cholangitis (PBC) are bile duct disorders with cholestatic liver injury (1). These cholangiopathies are characterized by ductular reaction, peribiliary inflammation, and liver fibrosis (2). During cholestatic liver injury, various hepatic cells proliferate to compensate for the damaged or lost cholangiocyte populations (3, 4). The first one involves cholangiocyte self-proliferation. Previous studies identified two distinct cholangiocyte phenotypes, large and small (5). Large cholangiocytes robustly proliferate as a response to biliary damage leading to ductular reaction (3, 4). Small cholangiocytes can differentiate into large cholangiocytes when large cholangiocytes are damaged *in vivo* (6–9). The second pathway is characterized by progenitor-derived cholangiocyte proliferation wherein liver stem cells, known as hepatic progenitor cells (HPCs), can differentiate into biliary phenotypes (10, 11). The last approach entails the transdifferentiation of hepatocytes. During severe biliary damage, hepatocytes transdifferentiate into cholangiocytes to compensate the damaged bile ducts (11, 12). Hepatic cells are heterogeneous and there are multiple phenotypes of cholangiocytes and hepatocytes. Previous studies demonstrated differences in functions and gene expressions between hepatocytes near the portal vein (Zone 1), those near the central vein (Zone 3), and those between Zone 1 and 3 (Zone 2) (13, 14). Single cell RNA sequencing (scRNA-seq) for liver tissues of deceased donors identified multiple cholangiocyte, HPC, and hepatocyte phenotypes with characteristic gene expression profiles (15, 16). However, the functional roles of different hepatic phenotypes in the pathophysiology of cholestatic liver injury are largely undefined. Ductular reaction in PSC and PBC is closely associated with peribiliary inflammation and liver fibrosis (4). Ductular reaction is an expanded reactive biliary phenotypes and can be detected by immunoreactivity against bile duct markers, such as cytokeratin 19 (CK-19, *KRT19*) or CK-7 (*KRT7*) (3). Although ductular reactive cells are biliary phenotypes, their origins can be cholangiocytes, HPCs, or hepatocytes (3). Functions of ductular reactive cells can differ depending on the origins of cells, but it is not feasible to determine the origins of ductular reactive cells by immunostaining.

Deconvolution methods are the computational techniques to calculate and estimate the abundance of different cell types from bulk transcriptome data (17). CIBERSORT is one of deconvolution methods (18), and CIBERSORTx is the updated version of CIBERSORT (19). CIBERSORTx can characterize cellular heterogeneity and gene expression profiles in specific cell types from bulk tissue transcriptomes, such as microarray data (19, 20). Previous studies compared multiple deconvolution methods and showed that both CIBERSORT and CIBERSORTx demonstrated robust and comprehensive performance in various experimental conditions compared to other deconvolution methods (21–24). CIBERSORTx performs deconvolution based on the reference data, which is referred to as signature matrix, and a signature matrix is created from scRNA-seq data (18, 19). CIBERSORTx has a default signature matrix file, LM22, which distinguishes 22 human hematopoietic cell phenotypes (18, 19). Previous studies performed deconvolution using CIBERSORT and LM22 to compare immune cell landscape between healthy liver and hepatocellular carcinoma tissues (25, 26). However, LM22 is only for immune cells and cannot identify hepatic cells, such as hepatocytes and cholangiocytes. CIBERSORTx allows users to create custom signature matrix using scRNA-seq data to perform deconvolution for specific cell phenotypes (19, 20).

It remains undefined whether a custom signature matrix for hepatic cell phenotypes can accurately estimate cell composition in bulk liver microarray data. Furthermore, there are no previous studies on performing deconvolution for hepatic cells in PSC and PBC. The current study aims to generate custom signature matrix files using liver scRNA-seq data and evaluate the effectiveness and reliability of CIBERSORTx with a custom signature matrix for hepatic cell phenotypes. To assess the accuracy of the cell composition estimated by CIBERSORTx, we compare it to established experimental data. For example, it is well known that intrahepatic bile duct mass is significantly increased during chronic liver diseases, such as cholangiopathies, which is referred to as ductular reaction (3). If cell deconvolution by CIBERSORTx successfully identifies elevated biliary phenotypes and ductular reaction in PSC and PBC livers, then CIBERSORTx has introduced a novel approach to estimate hepatic cell composition without performing cell sorting or scRNA-seq. In this study, we successfully applied the extensive performance of CIBERSORTx in deconvolution analysis for hepatic cell phenotypes and identified altered hepatic cell landscape in PSC and PBC liver tissues.

## 2 Materials and methods

### 2.1 Data collection

Transcriptome profiling data for human liver tissues were obtained from Gene Expression Omnibus (GEO, https://www.ncbi.nlm.nih.gov/geo/). GSE159676 contains microarray data of liver tissues for healthy controls (*n* = 6), patients with non-alcoholic steatohepatitis (*n* = 7), PSC (*n* = 12), PBC (*n* = 3), autoimmune hepatitis (*n* = 3), haemochromatosis (*n* = 1), and alcoholic liver disease (*n* = 1) (27). Data of controls, PSC, and PBC (total *n* = 21) were included in this study. GSE185477 contains scRNA-seq and single nucleus RNA-seq data of healthy liver tissues (15). scRNA-seq data generated from four liver tissues were used in this study. GSE115469 contains scRNA-seq data of five liver tissues (16), and all data were included in this study. GSE185477 and GSE115469 were used to create custom signature matrices, and GSE159676 was used as the target microarray data for deconvolution analysis.

### 2.2 Creation of custom signature matrix for CIBERSORTx

To create custom signature matrix files for CIBERSORTx, we selected GSE185477 and GSE115469 because: (i) original research articles for scRNA-seq data were available for characterization of each hepatic cell phenotype and associated gene profiles; (ii) processed data were available to download, and read counts were available (GSE185477) or information of normalization was available (GSE115469), since data normalization could impact the power of deconvolution in CIBERSORTx (19); (iii) scRNA-seq data were produced using cells of human fresh liver tissues; (iv) liver tissues were from neurologically deceased donors, and they were healthy individuals (GSE185477) or the authors in the original study confirmed normal histological patterns, so these livers were acceptable for liver transplantation (GSE115469). Therefore, these livers in both data series are suitable to be used as control livers; (v) both data series contain data of liver tissues from multiple deceased donors (*n* = 4 for GSE185477, *n* = 5 for GSE115469) to cover heterogeneous hepatic cell phenotypes in the liver; and (vi) both data series included all hepatic cells from total liver homogenates and did not select specific cell types, such as immune cells. Both used relatively a large number of hepatic cells to produce reliable hepatic cell atlas (73,295 cells in GSE185477, 8,444 cells in GSE115469) (15, 16).

R version 4.2.2 was used in this study. For GSE185477, we downloaded Seurat Objects containing read counts processed and uploaded by the authors from Dropbox^1^ using the Seurat package (28). GSE185477 contains data of single cell and single nucleus RNA-seq, and we used only scRNA-seq data. We extracted scRNA-seq data from the entire data, and gene and cluster annotations were used as the authors determined in their original study (15). Data were normalized and converted to transcripts per million (TPM) using the biomaRt package (29). For GSE115469, we downloaded processed and normalized data from GEO manually. Since data were in log2CPM (counts per million) format and CIBERSORTx suggests nonlog data (18, 19), we processed all data as: processed data = 2^(log2CPM values)^ – 1. We eliminated data of erythroid cells for custom signature matrix of GSE115469 because: (i) data of erythroid cells caused messy and unreliable output in deconvolution (data not shown); (ii) erythroid cells are minor cells in hepatic cell population; and (iii) GSE185477 does not classify any cells as erythroid cells, and elimination of erythroid cells from GSE115469 does not change consistency with GSE185477. For all data from GSE185477 and GSE115469, we downloaded data that the authors uploaded and used in their original studies (15, 16). It means that data set integration, quality control, and cell classifications and annotations were performed by the authors, and we did not change annotations and classifications that the authors determined. Total hepatic cells used in this study for generation of signature matrix files are 26,372 cells for GSE185477 and 8,351 cells for GSE115469.

The Docker version of CIBERSORTx^2^ was downloaded and used in this study. We created custom signature matrix files for processed data of GSE185477 and GSE115469 using the Fractions function of CIBERSORTx with following parameters: G.max, 50,000; replicates, 4 for GSE185477, 5 for GSE115469; fraction, 0; batch correction, B-mode. We performed B-mode batch correction for CIBERSORTx analysis because performance of B-mode is better than S-mode if a signature matrix file is available, as reported previously (30). Heatmaps for created signature matrix was generated by CIBERSORTx to visually check expression profiles for each hepatic cell phenotype.

### 2.3 Validation of custom signature matrix

We created data to be used for deconvolution (referred to as mixture files) for validation of custom signature matrix files. We shuffled GSE185477 and GSE115469 data, which were used for signature matrix creation, and selected data values of 3,000 cells randomly. We generated three validation mixture files for each signature matrix. For deconvolution to calculate cell composition from mixture files, we used CIBERSORTx Fractions with following parameters: permutations, 1,000; batch correction, B-mode.

### 2.4 Analysis of bulk microarray data using CIBERSORTx

Raw microarray data of GSE159676 in CEL formats were downloaded from GEO manually. We read CEL files and performed background correction and quantile normalization using the oligo package. Since microarray for GSE159676 was performed using Affymetrix Human Gene 1.0 ST Array, gene annotation was carried out using the hugene10sttrancriptcluster.db package. Data were exported in a text file, and we used it as the mixture file. We performed deconvolution for the GSE159676 mixture file using signature matrix of GSE185477 or GSE115469. CIBERSORTx Fractions were used with following parameters: permutations, 1,000; batch correction, B-mode. GSE185477 and GSE115469 classified hepatic cell phenotypes with characteristic marker gene expression identified in their studies (15, 16). CIBERSORTx calculates deconvolution *p*-values as quality control metrics, and fraction data with *p* < 0.0001 were used. Normalized read counts for these phenotype-associated marker genes were extracted from GSE159676 data to validate estimated cell composition with marker expression.

### 2.5 Statistical analysis

We performed Pearson correlation analysis between estimated cell population in percentage (fraction × 100) by CIBERSORTx and actual cell population in 3,000 cells using Prism version 9.5.1 (GraphPad Software, Boston MA). We also perform unpaired student’s *t*-test to compare cell fractions and marker gene expression levels between Healthy and PSC/PBC groups using Prism. Mean ± standard error of the mean (SEM) was plotted and differences with *p* < 0.05 were considered as statistically significant.

## 3 Results

### 3.1 Creation and evaluation of custom signature matrices for GSE185477 and GSE115469

**Figure 1:**
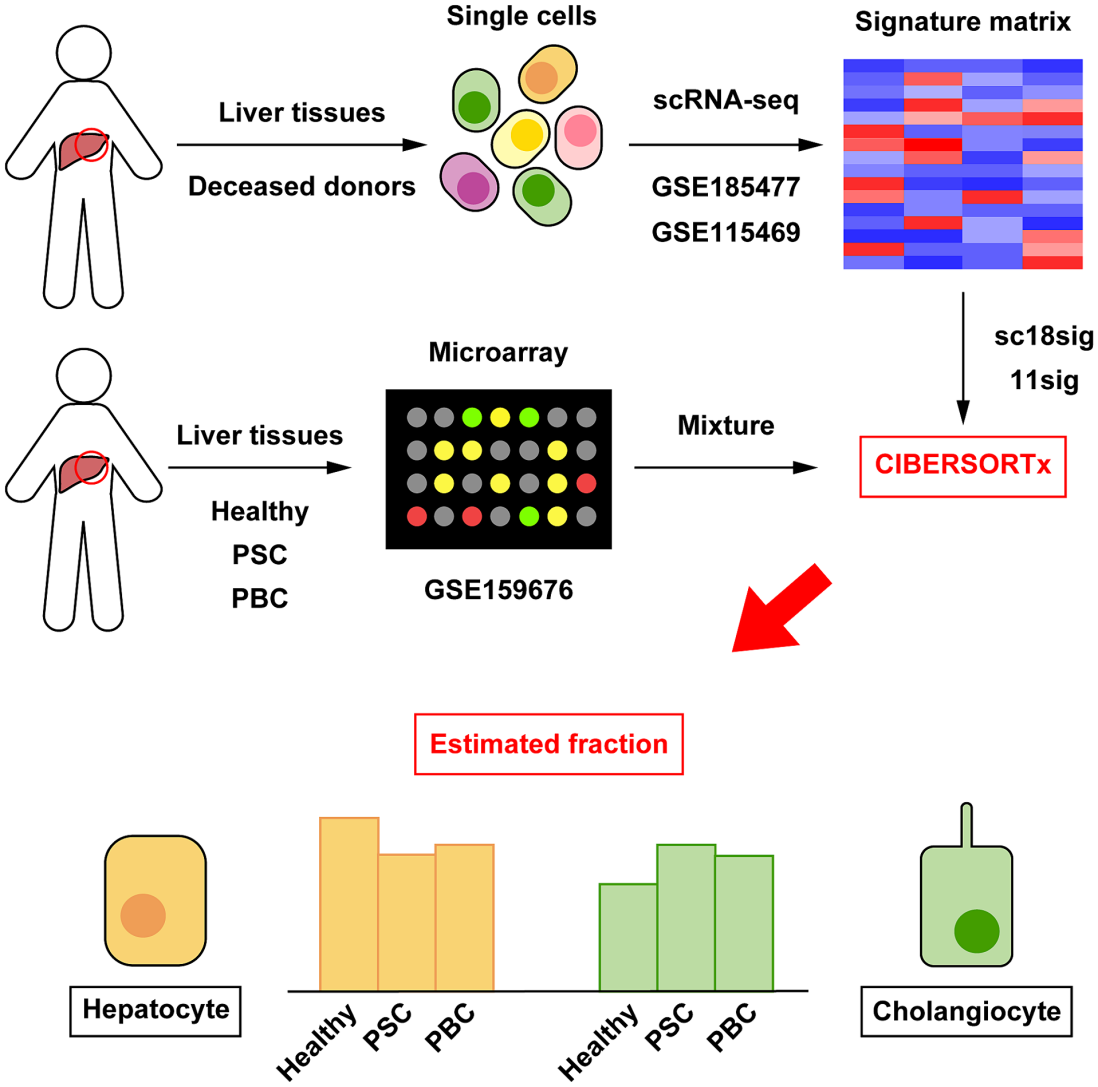
Outline of cellular deconvolution using CIBERSORTx in this study. Data of scRNA-seq (GSE185477 and GSE115469) were downloaded and used to create custom signature matrices (sc18sig and 11sig, respectively). Microarray data of human liver tissues (GSE159676) were downloaded and used as mixture, which is the target data for deconvolution. CIBERSORTx calculates cell composition in liver tissues of GSE159676 using sc18sig and 11sig.

**Figure 2:**
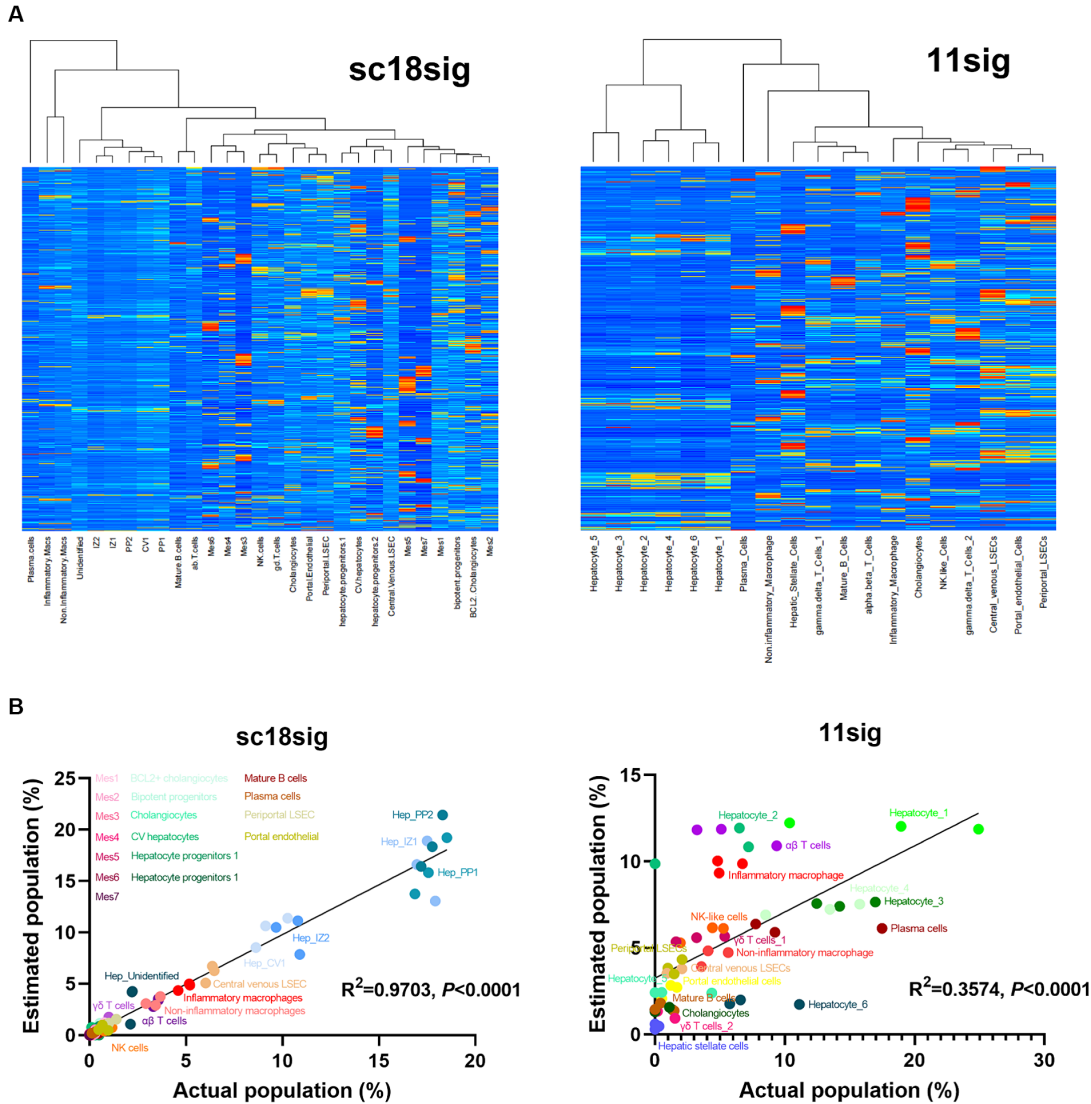
Characteristics of created custom signature matrices. Two signature matrices were created in this study: one using scRNA-seq data of GSE185477 (sc18sig), and one based on data of GSE115469 (11sig). **(A)** Heatmaps of sc18sig and 11sig generated by CIBERSORTx in the process of custom signature matrix creation. **(B)** Validation of sc18sig and 11sig. Deconvolution was performed using signature matrices and validation mixture files. Estimated cell fraction was multiplied by 100 to obtain estimated cell population, and Pearson correlation analysis was performed with actual population in validation mixture data.

CIBERSORTx calculates cell composition based on the signature matrix file, which is generated by scRNA-seq data (19). CIBERSORTx includes the default signature matrix, LM22, but LM22 is a leukocyte gene signature matrix designed to distinguish 22 human hematopoietic cell phenotypes and not suitable to distinguish hepatic cell phenotypes (18). This study aims to perform deconvolution using CIBERSORTx to estimate cell composition in liver tissues (Figure 1). We first generated custom signature matrix files using scRNA-seq data of human liver tissues. CIBERSORTx Fractions allows users to generate custom signature matrix and we successfully generated signature matrix files for GSE185477 and GSE115469, and these signature matrices are referred to as sc18sig and 11sig, respectively, in this study (Figure 2A). There are 29 hepatic cell phenotypes identified in sc18sig according to the original scRNA-seq study (15): *6 biliary phenotypes*—Cholangiocytes, B-cell lymphoma 2 (BCL2) + Cholangiocytes, Bipotent Progenitors, Hepatocyte Progenitors 1, Hepatocyte Progenitors 2, CV Hepatocytes; *6 hepatocyte phenotypes* – PP (periportal) 1, PP2, CV (central venous) 1, IZ (interzonal) 1, IZ2, Unidentified; *7 mesenchymal cell or hepatic stellate cell (HSC) phenotypes*—Mes1 to Mes7; *3 endothelial cell phenotypes*—Periportal LSEC (liver sinusoidal endothelial cell), Central Venous LSEC, Portal Endothelial; and *7 immune cell phenotypes*—αβ T cells, γδ T cells, Mature B cells, Plasma cells, Inflammatory Macs (macrophages), Non-Inflammatory Macs, NK (natural killer) cells. Nineteen hepatic phenotypes were identified in 11sig (16): *1 biliary phenotype*—Cholangiocytes; *6 hepatocyte phenotypes*—Hepatocyte_1 to Hepatocyte_6; *1 HSC phenotype*—Hepatic_Stellate_Cells; *3 endothelial cell phenotypes*—Periportal_LSECs, Central_venous_LSECs, Portal_endothelial_Cells; and *8 immune cell phenotypes*—αβ_T_Cells, γδ_T_Cells_1, γδ_T_Cells_2, Mature_B_Cells, Plasma_Cells, Inflammatory_Macrophage, Non-inflammatory_Macrophage, NK-like_Cells.

To validate custom signature matrix files, we performed deconvolution using CIBERSORTx Fractions with custom signature matrix and mixture files that were generated for validation. Both signature matrices show significant (*p* < 0.0001) correlation between estimated and actual cell population (Figure 2B), indicating that deconvolution using these signature matrices, especially in the case of GSE185477 (sc18sig), can estimate cell composition accurately in CIBERSORTx.

### 3.2 Cholangiocyte composition was increased in PSC/PBC liver tissues

**Figure 3:**
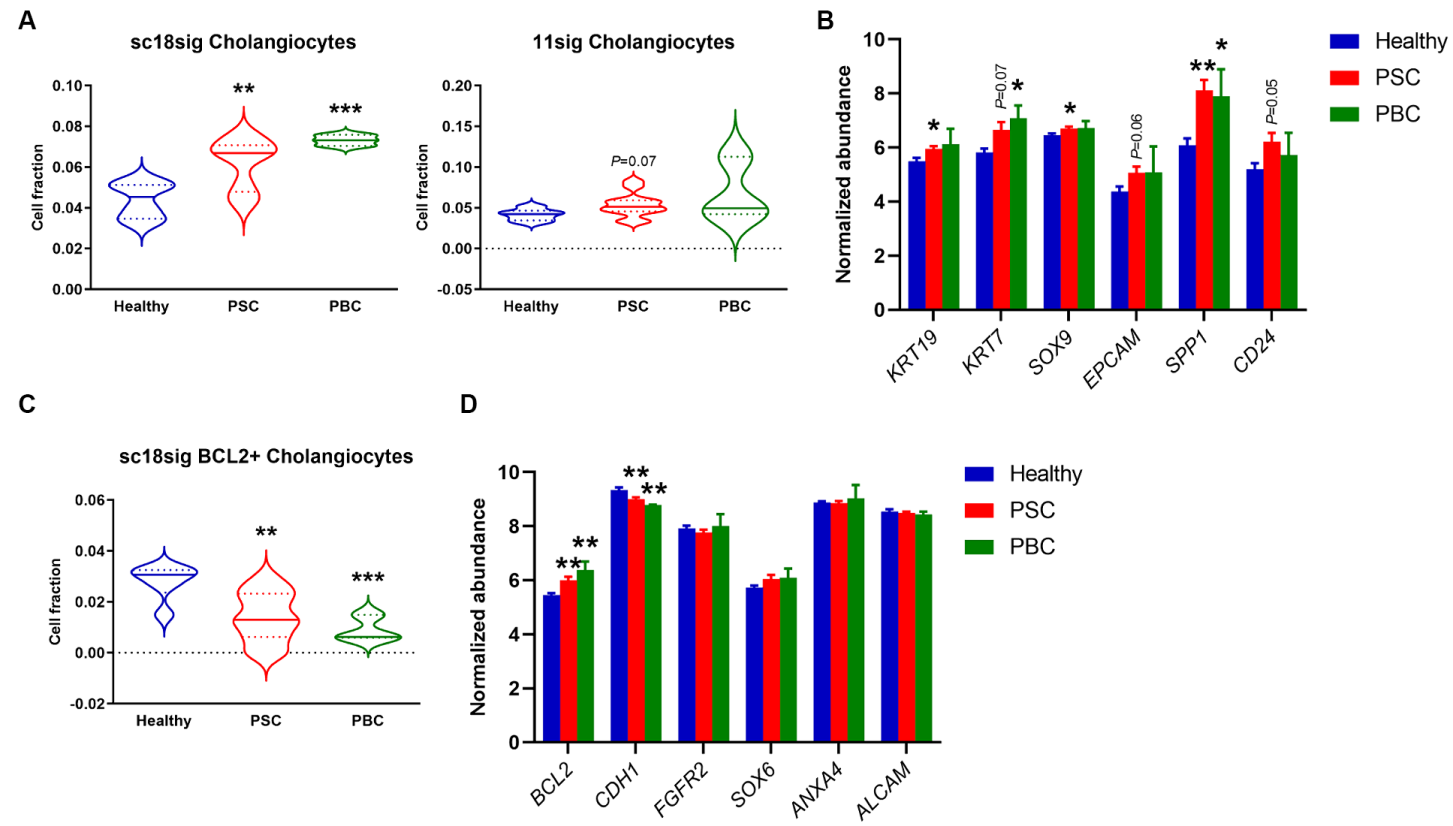
Cell composition for biliary phenotypes estimated by CIBERSORTx. Deconvolution was performed using CIBERSORTx with signature matrices, sc18sig and 11sig, and the mixture file of GSE159676. **(A)** Estimated fraction for the “Cholangiocytes” phenotype. **(B)** Microarray data of GSE159676 for genes associated with cholangiocytes. **(C)** Estimated fraction for “BCL2+ Cholangiocytes,” which are recognized as small cholangiocytes. **(D)** Expression for progenitor-associated genes in GSE159676. Mean ± SEM, ^*^p < 0.05, ^**^p < 0.01, and ^***^p < 0.001 vs. Healthy. Sample numbers are 7 for Healthy, 12 for PSC, and 3 for PBC.

We performed deconvolution to estimate hepatic cell phenotypes in healthy, PSC, and PBC liver tissues of GSE159676 using CIBERSORTx and our custom signature matrices. Both sc18sig and 11sig identified the elevated phenotype of “Cholangiocytes” in PSC and PBC livers compared to healthy liver tissues (Figure 3A). Ductular reaction is the expansion of CK-19+ or CK-7+ cells (i.e., cholangiocytes) commonly observed in PSC/PBC liver sections (3, 4). Therefore, elevation of cholangiocyte fraction in PSC/PBC livers was expected and identical to previous studies. Signature matrices identify the specific phenotypes with characteristic marker expression profiles (18, 19). For example, In GSE115469, the phenotype “Cholangiocytes” is recognized as cells with high expression of KRT7, KRT19, SRY-box transcription factor 9 (*SOX9*), and epithelial cell adhesion molecule (*EPCAM*), which are typical cholangiocyte markers (16). GSE185477 shows that cholangiocytes (Chol-4) are matured cholangiocytes with typical biliary marker expression, such as KRT7, as well as other markers such as osteopontin or secreted phosphoprotein 1 (*SPP1*) and CD24 (15). To confirm the estimated fractions by CIBERSORTx, we analyzed expression levels of these cholangiocyte markers in GSE159676 microarray data. Markers of mature cholangiocytes were significantly upregulated in PSC and PBC patients’ liver samples compared to healthy liver tissues (e.g., *KRT19*, *p* < 0.05 PSC vs. healthy, Figure 3B), supporting increased populations of cholangiocytes during cholestatic liver injury. In sc18sig, BCL2+ Cholangiocytes were significantly decreased in PSC/PBC liver tissues compared to healthy liver (*p* < 0.01 PSC vs. healthy and *p* < 0.001 PBC vs. healthy, Figure 3C). Previous studies demonstrated that small but not large cholangiocytes express BCL2 (31). Small cholangiocytes turn into large when the large is damaged, showing the progenitor cell-like features of small cholangiocytes (6–9). GSE185477 identified the BCL2+ cholangiocyte phenotype and demonstrated that these cells express progenitor-associated markers (15). Elevated composition of Cholangiocytes (large cholangiocytes) and decreased fraction of BCL2+ Cholangiocytes (small cholangiocytes) could be the result of transition of small cholangiocytes into large cholangiocytes during cholestatic liver injury. However, in GSE159676 data, expression levels of BCL2 were upregulated, not downregulated, in PSC/PBC livers (Figure 3D). For progenitor-associated markers, E-cadherin (*CDH1*) was significantly (*p* < 0.01) downregulated in PSC/PBC, but no difference was identified between groups for other progenitor markers (Figure 3D). Unlike CK-19 and CK-7 for large cholangiocytes, there are no specific markers that are extensively expressed in small cholangiocytes. Hepatocytes express BCL2 (32, 33), and the population of small cholangiocytes is relatively lower than that of hepatocytes in the liver. For instance, the populations of small cholangiocytes and hepatocytes including all phenotypes in sc18sig are 0.1 and 75.1%, respectively. Therefore, it is not feasible to confirm the results of CIBERSORTx by analyzing marker expression in GSE159676. Although there are other progenitor cell phenotypes in sc18sig (Bipotent Progenitors and Hepatocyte Progenitors 1/2), no significant difference in cell fractions or marker expression was identified in our study (data not shown).

### 3.3 HSC populations were increased in PSC/PBC liver tissues

**Figure 4:**
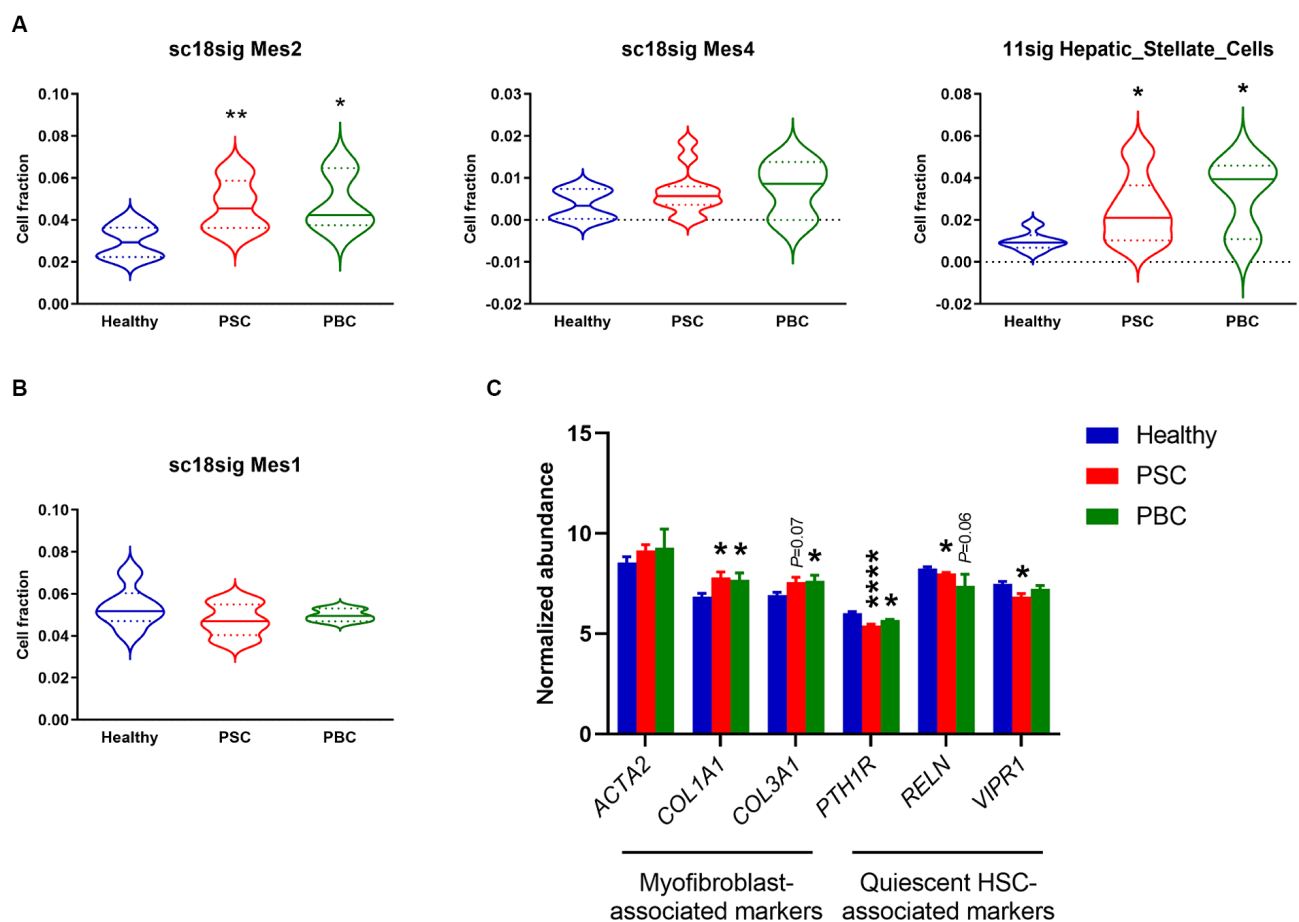
Cell composition for mesenchymal phenotypes estimated by CIBERSORTx. **(A)** Estimated fraction for myofibroblast-like HSCs (Mes2 and Mes4) using sc18sig and for “Hepatic_Stellate_Cells” using 11sig. **(B)** Estimated fraction for quiescent HSCs (Mes1) using sc18sig. **(C)** Expression for genes associated with quiescent and activated HSCs in GSE159676. Mean ± SEM, ^*^p < 0.05, and ^****^p < 0.0001 vs. Healthy (*n* = 7 for Healthy, *n* = 12 for PSC, *n* = 3 for PBC).

GSE185477 identified 7 mesenchymal cell phenotypes (Mes1-7) and showed that Mes2 and Mes4 were myofibroblast-like cells (15). Deconvolution using sc18sig showed elevated composition of Mes2 and Mes4 in PSC/PBC liver tissues, indicating increased populations of activated HSCs leading to liver fibrosis (Figure 4A). GSE115469 identifies only one mesenchymal cell phenotypes, Hepatic_Stellate_Cells (16). Elevated cell fractions for Hepatic_Stellate_Cells were observed in 11sig, supporting the increased HSC composition in PSC/PBC (Figure 4A). Mes1 is recognized as quiescent HSCs in GSE185477, and sc18sig exhibited decreased Mes1 composition in PSC/PBC livers, although data were not statistically significant (Figure 4B). Analysis of expression levels of HSC-associated markers in GSE159676 demonstrated that genes associated with myofibroblasts and hepatic fibrogenesis, such as collagen type I alpha 1 chain (*COL1A1*), were significantly (*p* < 0.05) upregulated in PSC and PBC liver tissues compared to healthy controls (Figure 4C). On the other hand, the markers associated with quiescent HSCs, such as parathyroid hormone 1 receptor (*PTH1R*), were significantly downregulated in PSC/PBC liver tissues (*p* < 0.0001 PSC vs. healthy and *p* < 0.05 PBC vs. healthy), indicating increased activation of HSCs leading to elevated fractions for myofibroblast type cells (Figure 4C). Ductular reaction is closely associated with activation of HSC and hepatic fibrogenesis in liver diseases (4). Activated and proliferative HSCs (myofibroblasts) are the major source of extracellular matrix secretion contributing to liver fibrosis, and their populations are increased during cholestatic liver injury (34). In addition to the increased fraction of large cholangiocytes, we observed the increased composition of activated HSCs, which are in agreement with previous studies. This consistency indicates the accuracy and reliability of the deconvolution analysis using CIBERSORTx.

### 3.4 Specific types of immune cells were increased in PSC/PBC liver tissues

**Figure 5:**
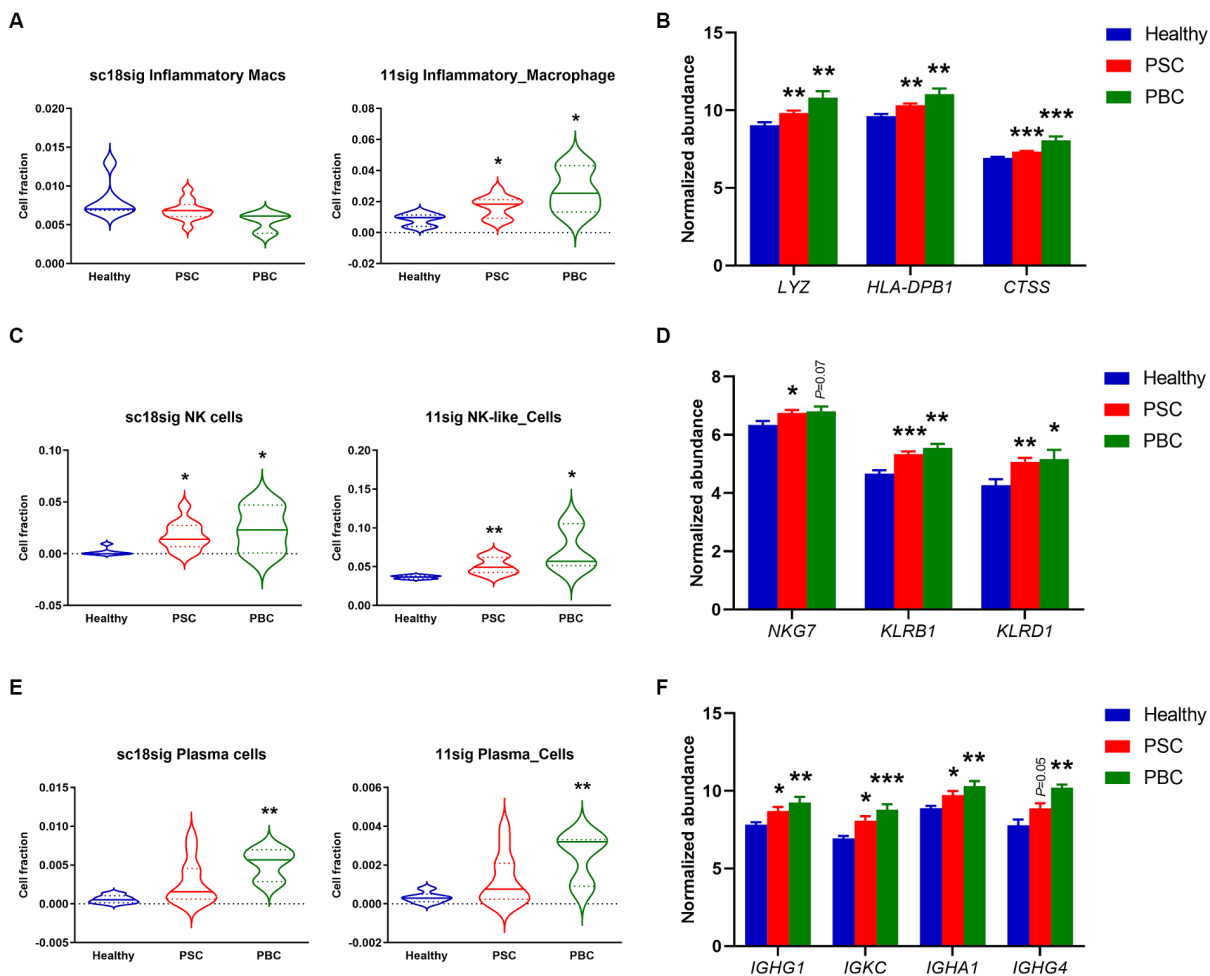
Increased fractions of specific immune cells estimated by CIBERSORTx. **(A)** Estimated composition for inflammatory macrophages using sc18sig and 11sig. **(B)** Expression levels of genes associated with inflammatory macrophages. **(C)** Estimated fractions for NK cells. **(D)** Marker expression associated with NK cells. **(E)** Estimated fractions for plasma cells. **(F)** Expression levels for plasma cell markers. Mean ± SEM, ^*^p < 0.05, ^**^p < 0.01, and ^***^p < 0.001 vs. Healthy (*n* = 7 for Healthy, *n* = 12 for PSC, *n* = 3 for PBC).

During cholestatic liver injury, bone marrow-derived macrophages infiltrate into the liver and macrophage population is increased compared to healthy livers (35, 36). Macrophages secrete proinflammatory cytokines leading to peribiliary inflammation, and population of inflammatory macrophages is increased in liver tissues of cholestatic model mice (37). In the current study, sc18sig did not identify significant differences between groups, but 11sig showed elevated population of inflammatory macrophages in PSC and PBC livers compared to healthy tissues (Figure 5A). Markers associated with inflammatory macrophages were significantly upregulated (*p* < 0.01 or *p* < 0.001) in PSC/PBC livers in GSE159676, supporting the increased fraction of inflammatory macrophages during cholestatic liver injury (Figure 5B).

A previous study demonstrated that natural killer (NK) cells were increased in the peripheral blood of PBC patients compared to control individuals (38). Protein expression in NK cells is altered during PSC, which is associated with functions and cytotoxic capacity of NK cells, indicating the functional roles of NK cells in cholestatic liver injury (39, 40). CIBERSORTx identified significantly (*p* < 0.05 or *p* < 0.01) elevated cell fractions for NK cells in PSC/PBC liver tissues compared to healthy livers, using both sc18sig and 11sig (Figure 5C). NK cell markers were also upregulated in PSC/PBC livers in microarray data (Figure 5D). These findings strongly support the hypothesis that NK cell population is increased during cholestatic liver injury, which could be associated with its pathophysiology.

Although previous studies indicated that plasma cells are increased in PSC and PBC, functional roles of plasma cells in cholestatic liver injury are still undefined (41–43). CIBERSORTx showed significantly (*p* < 0.01) increased plasma cell fractions in PBC with sc18sig and 11sig (Figure 5E). Although increased composition of plasma cells in PSC was not statistically significant (Figure 5E), markers for plasma cells were significantly (*p* < 0.05) upregulated in both PSC and PBC liver tissues compared to healthy controls (Figure 5F). These results indicate that specific immune cell phenotypes, inflammatory macrophages, NK cells, and plasma cells, are increased and could be involved in the pathogenesis of cholestatic liver injury.

### 3.5 Population of interzonal and periportal hepatocytes was decreased in PSC/PBC livers

**Figure 6:**
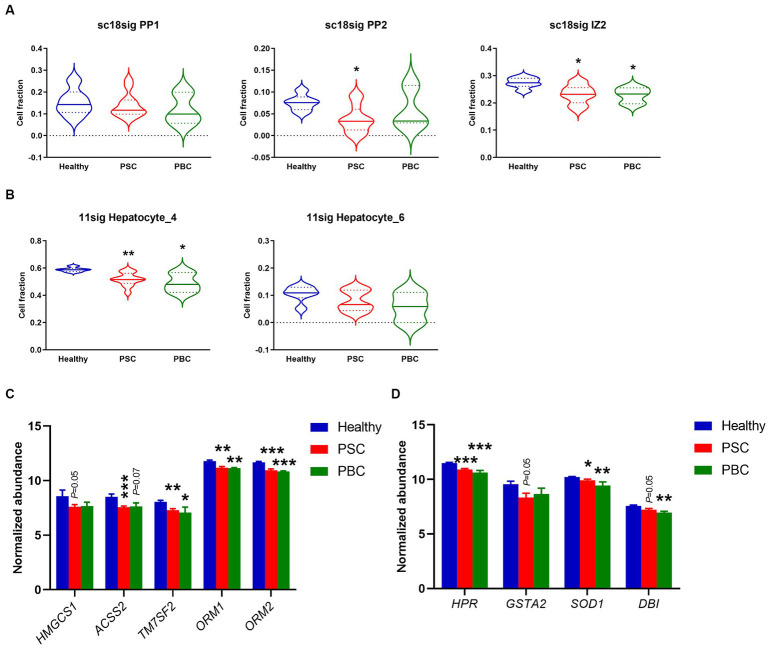
Decreased composition of periportal and interzonal hepatocytes in PSC/PBC livers. **(A)** Estimated composition for periportal and interzonal hepatocytes using sc18sig. **(B)** Estimated composition for periportal/interzonal hepatocytes using 11sig. **(C)** Marker expression associated with periportal hepatocytes. **(D)** Expression levels of marker genes for interzonal hepatocytes. Mean ± SEM, ^*^p < 0.05, ^**^p < 0.01, ^***^p < 0.001 vs. Healthy (*n* = 7 for Healthy, *n* = 12 for PSC, *n* = 3 for PBC).

Although previous studies suggested that protein expression is different in hepatocytes on different location (44, 45), functional roles of hepatocyte zonation in cholestatic liver disease are largely unknown. In the current study, sc18sig showed that hepatocyte phenotypes, PP1, PP2, and IZ2, which are classified as interzonal or periportal hepatocytes, were decreased in PSC and PBC liver tissues compared to Healthy control (Figure 6A). In GSE115469, Hepatocyte_4 (Cluster 6) is classified between periportal and interzonal, and Hepatocyte_6 (Cluster 15) is classified as interzonal hepatocytes (16). In 11sig, periportal/interzonal hepatocytes were significantly decreased in PSC (*p* < 0.01) and PBC (*p* < 0.05) livers, which is identical to results with sc18sig (Figure 6B). Markers associated with periportal hepatocytes and interzonal hepatocytes were significantly (*p* < 0.05) downregulated in the liver tissues of interest, supporting the reduced composition of periportal and interzonal hepatocytes during cholestatic liver injury (Figures 6C,D). We did not identify any differences between groups for central venous hepatocytes using sc18sig or 11sig (data not shown).

## 4 Discussion

**Figure 7:**
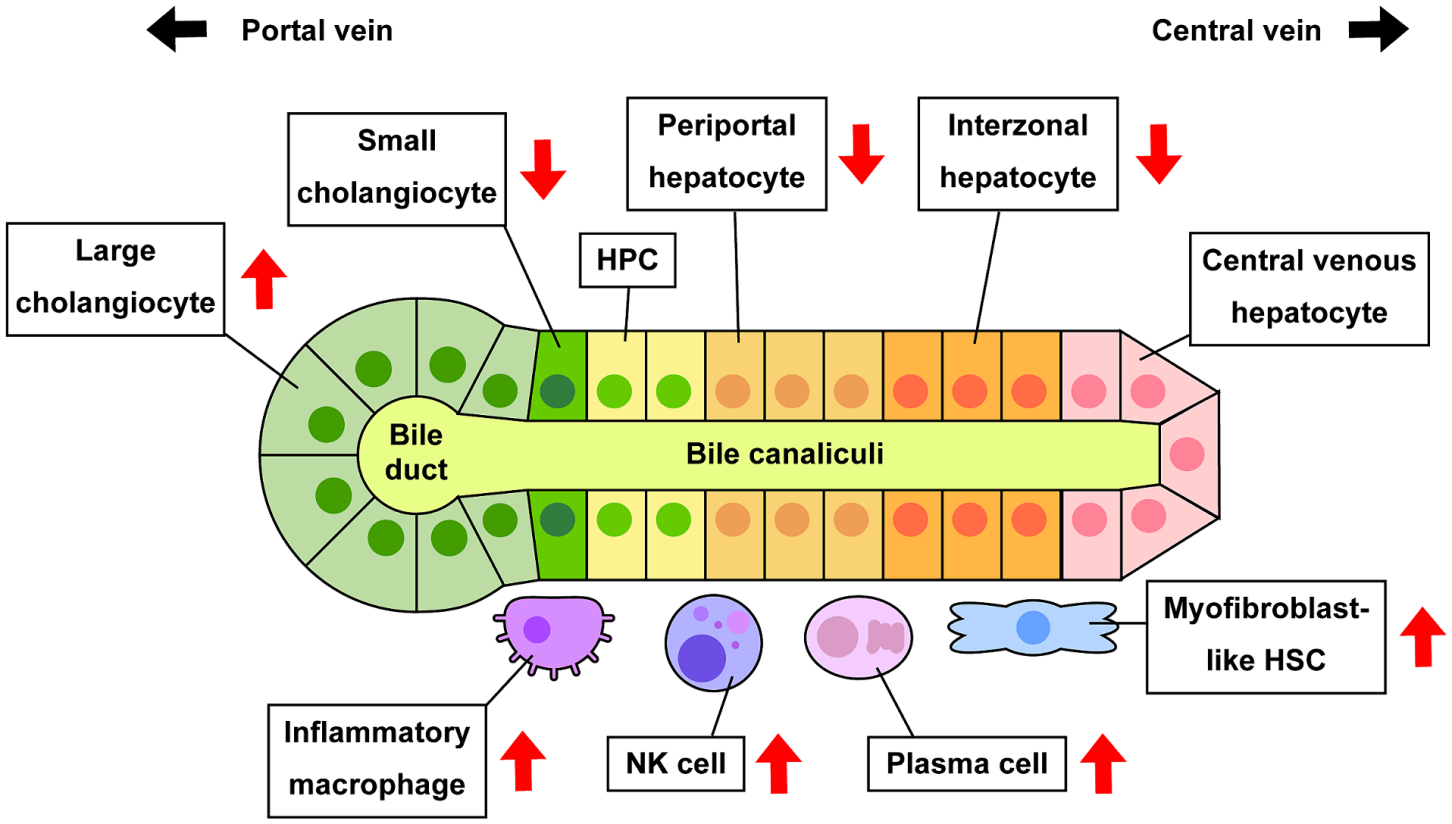
Alteration of cell composition during cholestatic liver injury. Previous studies and estimation using CIBERSORTx indicate that some specific hepatic cell phenotypes are increased or decreased in PSC and/or PBC compared to healthy conditions. Increased large cholangiocytes lead to ductular reaction, and increased activated HSCs and inflammatory macrophages lead to hepatic fibrogenesis or inflammation, respectively.

The current study successfully demonstrated extensive performance for deconvolution analysis to distinguish hepatic cell phenotypes in bulk microarray data using CIBERSORTx and custom signature matrices (Figure 7). This approach shows the potential of computational approach in estimation of cell composition in liver diseases. There are various cell deconvolution methods, and it is unclear which methods are suitable for hepatic phenotypes. It is also undefined whether deconvolution using CIBERSORTx with custom signature matrix can produce reliable and accurate results, consistent with experimental evidence using human or rodent liver tissues reported in previous studies. CIBERSORTx is one of the deconvolution methods that show reliable performance in various experimental conditions (21–24). In our study, we created two signature matrices, sc18sig and 11sig, and deconvolution results showed significant correlation with actual cell composition for both signature matrices, showing their extensive performance to estimate the population of each hepatic cell phenotype (Figure 2). Deconvolution results were consistent between sc18sig and 11sig in many cases, such as increased NK cells and HSCs in PSC and PBC livers. Although data processing for scRNA-seq, such as data integration, clustering, and annotation, differ between GSE185477 and GSE115469, results of deconvolution analysis were identical or similar between sc18sig and 11sig. These findings support the reliability of results in cell fraction estimation using CIBERSORTx and custom signature matrices. We validated the deconvolution results by analyzing expression levels of markers associated with specific hepatic cell phenotypes. Notably, the marker expressions were consistent with the deconvolution results (e.g., increased estimated NK cell composition and NK cell markers, such as NKG7, in PSC/PBC liver tissues). In addition, deconvolution results were in agreement with previous studies. For example, an increased population of large cholangiocytes was estimated and validated in this study, meanwhile proliferative large cholangiocytes were identified in cholestatic rats, which is now known as ductular reaction (4). These results strongly support that deconvolution using CIBERSORTx provides accurate estimation for cell composition of bulk liver microarray data without performing cell sorting or scRNA-seq. This approach can be useful to estimate cell composition for not only cholangiopathies but also for other liver diseases, such as metabolic dysfunction-associated fatty liver disease (MAFLD) (46) or steatohepatitis (MASH) (47). Since the current study is based only on computation using publicly available data series, scRNA-seq for PSC and PBC livers is required to determine actual hepatic cell composition during cholestatic liver injury. Our custom signature matrices were created using scRNA-seq data of control/healthy liver tissues, and cell clustering and annotations were depending on original studies (15, 16). GSE115469 identifies only one biliary phenotype and one HSC phenotype, “Cholangiocytes” and “Hepatic_Stellate_Cells,” respectively; therefore, 11sig can estimate cell fractions only for “Cholangiocytes” and “Hepatic_Stellate_Cells.” To estimate fractions of various hepatic cell phenotypes, it is required to use scRNA-seq data with more detailed cell classifications and annotations for signature matrix creation. To distinguish hepatic cells in diseased conditions, scRNA-seq data of PSC or PBC liver tissues may be required for custom signature matrix.

Identification of specific cell types is generally carried out by immunostaining targeting a marker (e.g., CK-19 for cholangiocytes); however, immunostaining cannot determine the origin of the cells. For example, ductular reaction can be identified by CK-19 staining, but it is not feasible to conclude if CK-19+ cells are self-proliferating large cholangiocytes, small-derived large cholangiocytes, or cholangiocytes differentiated from hepatocytes, only by immunostaining. To trace specific cell types and identify transdifferentiation during cholestatic liver injury, cell labelling techniques are required, such as adeno-associated virus (48, 49). Computation using CIBERSORTx estimates cell fraction in bulk microarray data and allows to speculate the roles of cell phenotypes. Our study demonstrated that large cholangiocytes were increased, but small cholangiocytes (BCL2+ cholangiocytes) and interzonal and periportal hepatocytes were decreased in liver tissues of PSC and PBC patients, compared to those from healthy individuals. Although this does not prove the transition of small cholangiocytes and interzonal/periportal hepatocytes into large cholangiocytes, these findings provide important implications: (1) there are multiple phenotypes in cholangiocytes and hepatocytes, and their functions could differ depending on the phenotypes; (2) PSC and PBC are bile duct disorders, but hepatocytes on the specific location may play a vital role in the pathophysiology of cholangiopathies; (3) experimental procedures should be carefully designed, as the analysis of the whole liver tissues or isolated cholangiocytes/hepatocytes including all phenotypes may potentially overlook critical findings. For example, if periportal hepatocytes, but not central venous and interzonal hepatocytes, are hypothesized to play an essential role in the pathogenesis of cholangiopathies, it may be necessary to design the experiments that specifically target periportal hepatocytes. In such cases, isolated hepatocytes from fresh liver tissues containing all cell phenotypes may not be appropriate. Therefore, cell sorting or laser-capture microdissection will be required in those cases.

CIBERSORTx is a powerful tool to estimate cell composition from bulk transcriptome data. CIBERSORTx performs deconvolution and fraction estimation using signature matrix, which is a critical data file for reliable results (19). LM22 is a default signature matrix provided by CIBERSORTx and it is used to estimate fractions of immune cells (18). Previous studies demonstrated deconvolution using CIBERSORT or CIBERSORTx revealing the immune cell landscape in liver tissues of patients with hepatocellular carcinoma or biliary atresia (25, 26, 50). However, this approach has certain limitations. For instance, LM22 was developed using data from peripheral blood mononuclear cells (18), and immune cells in the liver may exhibit different gene expression profiles; therefore, the deconvolution analysis of hepatic cells using LM22 may lack accuracy and reliability. In our study, we performed deconvolution for GSE159676 mixture using CIBERSORTx and LM22 but could not obtain consistent data, such as increased macrophage population (data not shown). Since portal infiltration and increased macrophage population during cholestatic liver injury are well known phenomena (35), this indicates that LM22 cannot estimate hepatic cell composition in the GSE159676 liver microarray dataset. In addition, LM22 is only for immune cells, and custom signature matrix is required to estimate fractions of different cell phenotypes, such as cholangiocytes. Our approach utilizing sc18sig and 11sig demonstrated extensive deconvolution performance, yielding results that align consistently between the two signature matrices and with experimental findings reported in the literature. Our study is the first to underscore the promising potentials of deconvolution analysis for estimation of hepatic cell phenotypes in cholestatic liver tissues.

Our approach using CIBERSORTx has certain limitations and weaknesses: (i) we analyzed GSE159676 data as mixture, but sample numbers are relatively low, especially for PBC patients (*n* = 6 for healthy control, *n* = 12 for PSC, and *n* = 3 for PBC). Differences between groups were not statistically significant in some cases, probably due to small sample size; (ii) it is hard to validate results obtained from microarray data for minor cells. Expression levels of genes associated with interzonal and periportal hepatocytes were decreased in microarray data of GSE159676, supporting the decreased fractions of these hepatocyte phonotypes. However, small cholangiocytes are very minor hepatic cells (only 0.1% in sc18sig), microarray data for genes associated with small cholangiocytes, such as BCL2, were not consistent to CIBERSORTx results. Further studies are required to elucidate the roles of small cholangiocytes and the transition into large cholangiocytes during cholestatic liver injury; (iii) there are certain hepatic cell phenotypes that remain unidentified by this approach. For example, previous studies demonstrated that mast cells play a key role in the pathogenesis of PSC, and mast cell numbers are increased in PSC liver sections (51). However, both GSE185477 and GSE115469 did not classify any cells as resting or activated mast cells, and hence sc18sig and 11sig were unable to provide estimates of mast cell fractions in the GSE159676 dataset; and (iv) this approach is not suitable for tumor tissues because both signature matrices are generated using control liver data. A new signature matrix file is needed to be generated from scRNA-seq data of liver tissues with tumors to distinguish normal and malignant hepatic cells. In addition, PSC is a common risk factor for cholangiocarcinoma (CCA) (52), but detailed mechanisms of biliary tumorigenesis mediated by cholestatic liver injury are still undefined. Therefore, there may be novel hepatic cell phenotypes in PSC livers, such as a precursor phenotype of CCA, and our approach in this study may not be able to distinguish these phenotypes from normal cholangiocytes. Our approach may not be suitable for liver tissues of late stage PSC or early stage CCA.

The current study focuses on PSC and PBC, but our approach can also be applied to other liver diseases or conditions. However, specific hepatic cell phenotypes may emerge only during the diseased conditions or may be very rare in normal liver tissues, making their identification challenging in scRNA-seq. For example, previous studies demonstrated that activated HSCs and portal fibroblasts contribute to hepatic fibrogenesis during cholestatic liver injury (53, 54). GSE185477 used healthy liver tissues, and identified 7 mesenchymal phenotypes, Mes1 to Mes7 (15). The authors referred to Mes2 and Mes4 as myofibroblast-like cells, but it is unclear if they are activated HSCs or portal fibroblasts. To generate sc18sig, data of total 26,372 hepatic cells were used, and 65 cells (0.2%) were identified as Mes2, and 17 cells (0.06%) were Mes4. Only 4–8 cells (0.01–0.03%) were found in sc18sig as Mes3, 5, 6, and 7, suggesting that healthy liver tissues may not naturally contain substantial numbers of mesenchymal cells. Consequently, a signature matrix created from healthy livers may not be effective in distinguishing between various mesenchymal phenotypes due to their low numbers under healthy conditions. Hepatic macrophages have two origins, Kupffer cells and bone marrow-derived macrophages, and multiple phenotypes, such as M0, M1, and M2 subsets (35, 55, 56). However, both GSE185477 and GSE115469 can identify only two macrophage phenotypes, non-inflammatory and inflammatory. Therefore, sc18sig and 11sig were unable to estimate the fractions for M0, M1, or M2 subsets or distinguish their origins. Although it is essential to create a signature matrix encompassing various macrophage phenotypes and origins, achieving this using healthy liver tissues may not be feasible because some phenotypes may not emerge in the normal conditions. It is also possible that there could be novel biliary or hepatocyte phenotypes during cholestatic liver injury that are not yet known. Ductular reaction is characteristic in cholangiopathies, such as PSC, and is mediated by proliferative and reactive biliary phenotypes leading to the expansion of intrahepatic bile ducts (3, 4). However, elevated cellular senescence is also commonly identified in biliary phenotypes in PSC (57, 58), which is closely associated with liver fibrosis in cholangiopathies (59, 60). It is possible that there may be different biliary phenotypes only present in cholestatic liver tissues, such as proliferating cholangiocytes and senescent cholangiocytes, and these biliary phenotypes may not be found in healthy livers. Deconvolution using sc18sig and 11sig may miss these disease-specific hepatic phenotypes. Further studies involving scRNA-seq for diseased liver tissues, such as PSC/PBC livers, are required to identify all hepatic cell phenotypes during liver injury and comprehensively assess the cell composition for each phenotype in the liver at diseased states.

In conclusion, the current study demonstrated that population of specific hepatic cell phenotypes was altered during PSC or PBC, which may be associated with the pathophysiology of cholangiopathies. Further studies are required to elucidate the detailed roles of these phenotypes in cholestatic liver injury.

## Data availability statement

Publicly available datasets were analyzed in this study. This data can be found at: GSE159676, GSE185477, GSE115469.

## Ethics statement

Ethical approval was not required for the studies involving humans because this study used human datasets uploaded in the public database. The authors did not have or use any human subjects or samples. The studies were conducted in accordance with the local legislation and institutional requirements. Only transcriptome data downloaded by the public database were used. Written informed consent to participate in this study was not required from the participants or the participants’ legal guardians/next of kin in accordance with the national legislation and the institutional requirements.

## Author contributions

HP: Writing – review & editing. LP: Writing – review & editing. KS: Writing – original draft, Project administration, Methodology, Conceptualization.

## Footnotes

^1^ https://www.dropbox.com/sh/sso15ehqmrrh6mk/AACKHOsSlZW0_Zy9cbCkOmMfa?dl=0

^2^ https://cibersortx.stanford.edu/
